# Correlation Between the Revised Physiological Trauma Score and CT Scan Results

**DOI:** 10.7759/cureus.67534

**Published:** 2024-08-22

**Authors:** Maan Jamjoom, Faisal A Boker, Ahmad Wazzan, Moath M Alshanqity

**Affiliations:** 1 Emergency Medicine, Ministry of the National Guard-Health Affairs, Jeddah, SAU; 2 Emergency Medicine, King Abdullah International Medical Research Center, Jeddah, SAU; 3 Emergency Medicine, King Saud Bin Abdulaziz University for Health Sciences, Jeddah, SAU

**Keywords:** multidetector computed tomography, prehospital trauma, clinical decision rule, medical triage, major trauma

## Abstract

Background

Trauma-scoring systems are used to triage patients and assist in clinical decision-making. Physiological trauma scores are used for quantitative evaluation of injury severity. However, only a few, such as the Revised Physiological Trauma Score (rPTS), have been proven effective in pre-clinical use. There is a constant need for clinical decision tools that aim to reduce the unnecessary use of CT scans among trauma patients. To our knowledge, no study has directly correlated the rPTS and CT findings. This study aimed to investigate whether the rPTS is correlated with CT scan results and can be used to decrease the use of CT.

Methodology

This retrospective chart review examined all patients who underwent a pan-CT for trauma in the Emergency Department of King Abdulaziz Medical City, Jeddah, from 2008 to 2012.

Results

We analyzed 235 patients. There was a significant difference in the mean rPTS between those with negative versus positive pan-CT scans (11.4 ± 1.3 vs. 10.9 ± 1.7, respectively; p = 0.032). Furthermore, the rate of positive CT scans was significantly higher in those with an rPTS <11 than those with an rPTS of 11 or 12 (87% vs. 74.1%, respectively; p = 0.044). However, 72.7% of patients with an rPTS of 12/12 had a positive pan-CT scan.

Conclusions

Despite the difference in the frequency of abnormal CT scans, too many patients with normal rPTS had abnormal CT findings. Therefore, the rPTS cannot be used to safely reduce the use of CT scans.

## Introduction

Accidental injury is one of the leading causes of death worldwide. Annually, there are approximately 4.4 million injury-related deaths, and one in three of these deaths are a result of moving vehicle collisions (MVCs) [1]. In Saudi Arabia, MVC is the main cause of trauma. The 2018 WHO Global Status Report on Road Safety found that in 2016, the mortality rate was 28.8 deaths per 100,000 population, with a total of 9,311 fatalities [2,3].

CT scans are a cornerstone of the evaluation of high-risk trauma cases, but their use and overuse contribute greatly to the burden of trauma on healthcare systems that are already strained [4,5]. This high burden has encouraged the search for tools that can reduce the unnecessary use of CT scans. For example, the National Emergency X-Radiography Utilization Study (NEXUS) chest decision criteria have been validated for ruling out the need for chest CT among trauma patients [6,7].

Another set of tools that are being used and studied are trauma scores, which are systems that help stratify risk based on different easily assessed variables [8,9]. This study focuses on the use of the Revised Physiological Trauma Score (rPTS). The rPTS has been proven to be effective in preclinical use and is widely utilized to quantitatively evaluate injury severity [10]. However, to our knowledge, no previous study has examined the correlation between the rPTS and positive CT findings. In this study, we aimed to identify whether a normal physiological score plays a role in predicting negative pan-CT scan results in trauma patients.

## Materials and methods

Ethical approval was obtained from King Abdullah International Medical Research Center (approval number: IRB/0996/24). Our review included trauma patients who presented to the King Abdulaziz Medical City-Jeddah (KAMC-J) Emergency Department (ED) from 2008 to 2012. This is a retrospective, chart review, cross-sectional study. The data of 315 trauma patients were collected; however, due to missing data, only 235 patients were included in the analysis. Data were gathered and organized by our investigators.

We included all blunt trauma patients who presented to KAMC-J and underwent a pan-CT scan. We excluded those who had penetrating injuries, who did not undergo a pan-CT scan, those evaluated in another facility before presenting to our ED, and those with incomplete data at presentation such as pre-intervention vitals.

The following data were collected from each patient: trauma score components for which a score of 0 to 4 is given (Glasgow Coma Scale, systolic blood pressure, and respiratory rate); the combined score is a sum of those scores out of 12 (Appendices). Additional data gathered included heart rate and random blood glucose at presentation. We considered patients with a score of less than 11 as abnormal (a commonly used cut-off in determining the need for trauma center care in the prehospital setting) [11]. We considered any abnormal finding on a pan-CT to be a positive CT scan. This included but was not limited to evidence of traumatic brain injury as well as skull, vertebral, facial, pelvic, and rib fractures. In addition to pneumothorax, hemothorax lung contusions and liver and spleen lacerations were observed.

After the data were collected, they were entered into Microsoft Excel (Microsoft Corp., Redmond, WA, USA). Data analysis was conducted using SPSS Statistics version 23.0 (IBM Corp., Armonk, NY, USA). We described quantitative variables using means and standard deviations. We described qualitative variables using percentages and frequencies. To compare the frequency of positive CTs among those with low and high rPTS, we used Student’s t-test. We considered p-values <0.05 statistically significant.

## Results

The patients were divided into two groups based on their rPTS (Table 1). Patients with an rPTS <11 had abnormal CT findings significantly more frequently than those with an rPTS of 11 or 12 (74.1% vs. 87%, respectively; p = 0.044). Furthermore, the two groups differed significantly in the frequency of liver laceration (21.7% vs. 9%), intracranial injury (54.3% vs. 22.8%), skull fractures (15.2% vs. 4.8%), and vertebral fractures (37% vs. 20.1%). For those with an abnormal rPTS, the odds ratio for having an intracranial injury was 4 (confidence interval = 2.1-7.9, p < 0.01). Approximately 73% of patients with a normal rPTS had an abnormal CT scan.

**Table 1:** Study sample grouped by Revised Physiological Trauma Score (rPTS). Comparison of vitals, CT findings, and fractures. P: Chi-square for frequency comparison and t-test for mean comparison. ^*^: components of rPTS; ^†^: p < 0.05; ^¥^: other fractures include ribs, clavicle, scapula, and sternal fractures.

	rPTS ≥11 (n = 189)	rPTS <11 (n = 46)	P-value
Vitals	Mean ± SD		
Systolic blood pressure^*^	129 ± 20.5	121 ± 37.7	0.18
Respiratory rate^*^	22.2 ± 3.8	20 ± 7.7	0.17
Glasgow Coma Scale^*^	14.2 ± 1.7	5.6	<0.01^†^
Heart rate	110 ± 23.3	110 ± 32	0.06
Random blood glucose (mmol)	8.5 ± 2.9	8.9 ± 3	0.47
CT results	Frequency (%)		
Any abnormal CT finding	140 (74.1 %)	40 (87%)	0.044^†^
Pneumothorax	27 (14.3%)	9 (19.5%)	0.25
Lung contusion	15 (7.9%)	5 (10.9%)	0.35
Liver laceration	17 (9%)	10 (21.7%)	0.02^†^
Spleen laceration	16 (8.5%)	4 (8.7%)	0.58
Intracranial injury	43 (22.8%)	25 (54.3%)	<0.01^†^
Any fracture	98 (51.9%)	30 (65.2%)	0.07
Vertebral fracture	38 (20.1%)	17 (37%)	0.02^†^
Pelvic fracture	9 (4.8%)	4 (8.7%)	0.24
Facial fracture	34 (18%)	8 (17.4%)	0.56
Skull fracture	9 (4.8%)	7 (15.2%)	0.02^†^
Other fractures^¥^	44 (23.3%)	14 (30.4%)	0.2
Other findings	19 (10.1%)	11 (23.9%)	0.02^†^

**Figure 1:**
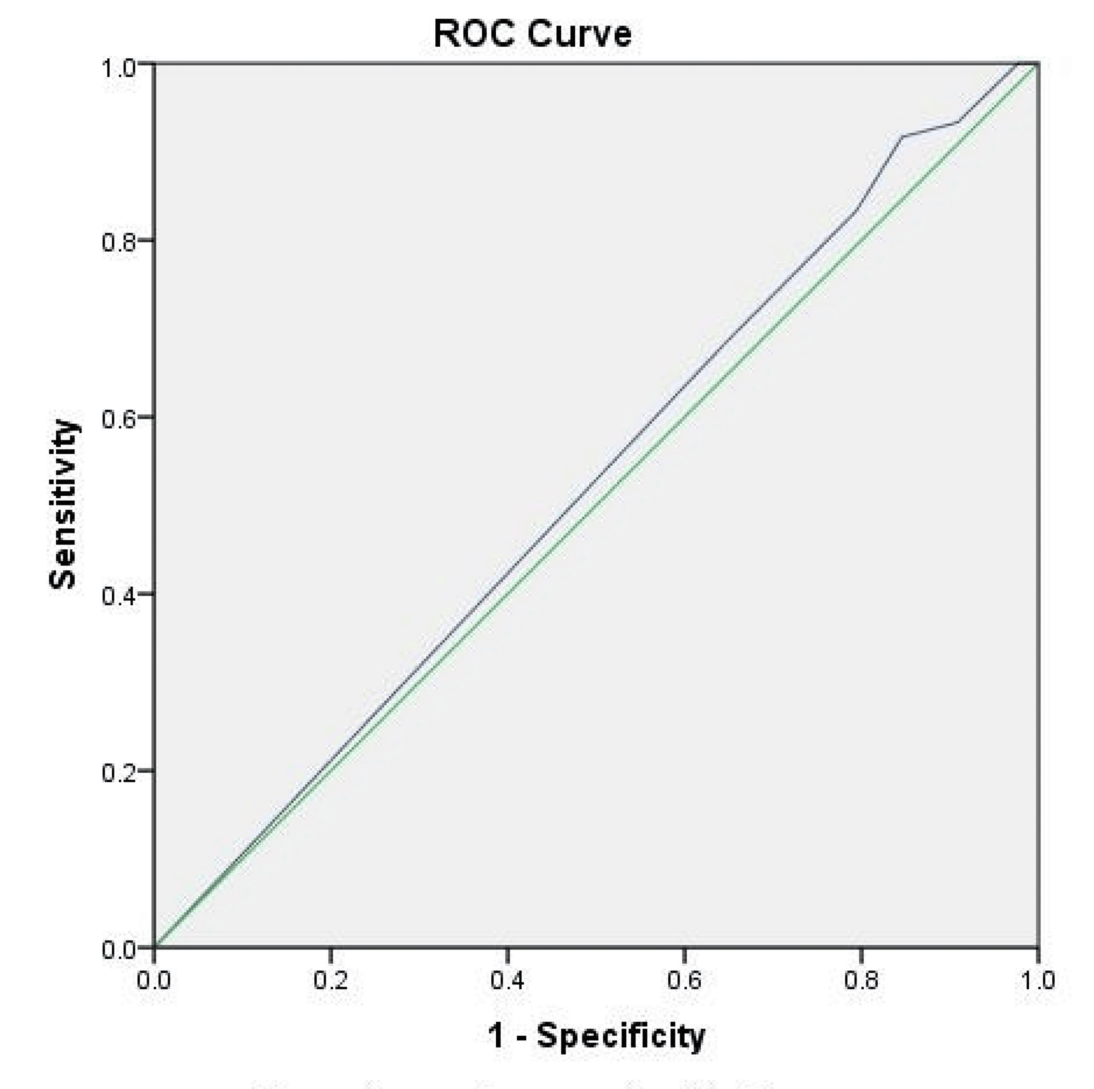
Receiver operating characteristic curve (ROC) of the predictive value of the Revised Physiological Trauma Score (rPTS) for negative pan-CT scans across different cut-off scores. The diagonal line (green) is produced by ties. The area under the curve is 0.526.

Furthermore, to determine the overall accuracy of the rPTS for predicting abnormal CT scans across different cutoff scores, a receiver operating characteristic curve was plotted. The rPTS had an area under the curve (AUC) of 0.52 for detecting abnormal CT scans (Figure 1).

## Discussion

This study investigated the utility of a commonly obtained and easily measured score. However, despite the statistically significant difference in the proportion of positive CTs between the two groups, the fact that approximately 73% of patients with a normal rPTS still had a positive CT scan limits the clinical utility of this tool. This is further demonstrated by the relatively low AUC value of 0.52. The ability of the trauma score to detect those with intracranial injuries or skull fractures can be expected given that a patient’s Glasgow Coma Scale score is a component of the rPTS [12]. However, according to our study and previous research, neither score is sufficient to exclude the possibility of intracranial injury alone [13]. One study found that in a group of 75 nonagenarians who required intervention for chronic subdural hematoma, more than 90% of patients had a Glasgow Coma Scale score of 13-15 upon presentation [14]. Therefore, the combination of Glasgow Coma Scale score or rPTS along with any other applicable scoring systems may be more applicable to stratify brain injury levels [15].

A drawback of our study was that the outcome of interest was the mere presence or absence of any CT finding, which is not a patient-centered outcome. Future research should investigate the ability of rPTS or other scores to predict surgically relevant CT findings or other findings that alter patient management. On the other hand, recent studies have already demonstrated that the sensitivity of trauma scores in predicting mortality, specifically Revised Trauma Score is 100%, with an AUC value of 0.957 [16].

## Conclusions

In summary, when blunt trauma patients arrive at the ED with decreased rPTS, they are more likely to exhibit abnormal CT scans, especially in cases of intracranial injuries or skull fractures. However, relying solely on a normal rPTS is insufficient to exclude abnormal CT findings, and it should not be the sole basis for forgoing CT scans. Future research could explore whether combining rPTS with other relevant systems improves risk stratification. Additionally, investigating whether similar scores, beyond mortality prediction, aid in identifying relevant CT findings for surgical decisions or management adjustments would be valuable.

## Appendices

**Table 2:** Revised Trauma Score calculation and scoring. Each of the three valuables is assessed and their coded values are summed to calculate the Revised Trauma Score (out of 12). A score of less than 12 is considered low/abnormal.

Glasgow Coma Scale score	Systolic blood pressure	Respiratory rate	Coded value
13–15	>90	10–29	4
9–12	76–89	>30	3
6–8	50–75	6–9	2
4–5	1–49	1–5	1
3	0	0	0
